# Within the Hospitals: The London

**Published:** 1913-12-06

**Authors:** 


### WITHIN THE HOSPITALS.

### The London Hospital.

### PROPOSED TUBERCULOSIS DISPENSARY.

NEGOTIATIONS are proceeding with the London County Council and the Local Government Board for the establishment of a tuberculosis dispensary for the treatment in one special department of the various forms of tuberculosis. The London Hospital, with specialists daily in attendance and with a staff of bacteriologists, pathologists, and radiographers, is the centre for the treatment of tuberculosis, and negotiations are already pending to co-operate in working with other dispensaries in the district.

### A SPECIAL DEPARTMENT FOR SYPHILIS.

We are informed that the London Hospital, owing to the personal acquaintance of Dr. Bulloch, its bacteriologist, with Professor Ehrlich, to whom the profession owe salvarsan, was the first institution in England in which the latter's treatment was used. A report of a special sub-committee of the London Hospital on the possibility of cure by salvarsan shows that now there is a probability of eradicating this disease. To make the treatment effective, each patient must be under observation in the hospital for a short time. On the recommendation of this sub-committee a department with certain reserved beds is in course of formation, for which the plans have been prepared, at an estimated cost of £10,500. To enable this work to be undertaken promptly the committee hope for the financial aid of some individual or public body.

### MATERNITY ALMONER AND NATIONAL INSURANCE.

The London Hospital treats maternity patients in their own homes, providing they are within a mile radius of the hospital. The Insurance Act, under which each insured person receives 30s., has placed the system of maternity letters hitherto in force on a new footing. With the new year a maternity almoner will be made responsible for the giving out of maternity letters, and will be charged with the duty of entering into an agreement with each patient as to the proportion of insurance benefit which should be handed over to the hospital as a contribution.

### THE EVER-INCREASING DEMAND FOR BEDS AND SPACE.

Owing to the advancement of medical science and to overwhelming pressure of work recently, the space available for the following departments at the London Hospital has proved inadequate. The clinical laboratory, the department in which chemical and other tests are made to determine or assist in diagnosis, has become an absolutely indispensable adjunct to hospital treatment. *X*-ray work has advanced enormously in recent years, and is now much used in the diagnosis of many and various complaints, such as lung and heart affections and cancerous growths. The cardiographic department, established under the direction of the eminent heart specialist, Dr. James MacKenzie, with Dr. John Parkinson as assistant, is doing excellent work in the treatment and investigation of heart complaints, but is also much hampered by lack of space. In this department investigations are being made into the causes of heart affections resulting from rheumatic fever. This is a most important branch of work, since one of the greatest curses of the East End is rheumatic fever, which attacks young children and leaves the heart so impaired that they are incapable of any strain in after life. The work of the registrars is that of collating the notes of all patients in the hospital, and of acting as substitutes for members of the staff. Eight years ago the number of registrars was two. It is now nine, and the space available for their offices is only the same as it was when their number was two. The Marie Celeste Samaritan Society has also petitioned for enlargement of its quarters. The work of this Society necessitates the interviewing of a number of patients with a view of assisting in sending them to convalescent homes or obtaining surgical appliances. The restricted space for the large number of patients which often have to be interviewed renders enlargement necessary.

